# Susceptibility and lack of evidence for a viremic state of rabies in the night owl monkey, *Aotus nancymaae*

**DOI:** 10.1186/1743-422X-9-95

**Published:** 2012-05-21

**Authors:** Erik J Reaves, Gabriela Salmón-Mulanovich, Carolina Guevara, Tadeusz J Kochel, Thomas J Steinbach, David E Bentzel, Joel M Montgomery

**Affiliations:** 1 Naval Medical Research Unit No. 6, Av. Venezuela cdra. 36 s/n, Callao 2, Peru; 2 Walter Reed Army Institute of Research, 503 Robert Grant Av, Silver Spring, MD, 20910-7500, USA

**Keywords:** Rabies, Rhabdovirus, Lyssavirus, Incubation period, Viremia, Monkey, *Aotus nancymaae*, Non-human Primate, *Ante mortem*, Vaccine

## Abstract

**Background:**

Rabies causes an acute fatal encephalomyelitis in most mammals following infection with rhabdovirus of the genus Lyssavirus. Little is known about rabies virus infection in species of New World non-human Primates (NHP). To investigate the suitability of the owl monkey *Aotus nancymaae* asissue sections examined were unremarkable for inflammation or other histologic signs of rabies a viable animal model for rabies virus candidate vaccine testing, we used clinical presentation, serology, viral isolation, and PCR to evaluate the incubation period, immunity, and pathogenesis of infected animals. We tested the hypothesis that no viremic state exists for rabies virus.

**Methods:**

Eight monkeys divided into two equal groups were inoculated intramuscularly either in the neck or footpad with 10^5^ pfu of rabies virus (Pasteur/V-13R) and observed for >130 days. Oral and blood samples were collected and analyzed.

**Results:**

Two monkeys inoculated in the neck displayed classic paralytic rabies. The mean incubation period was 11.5 days. The average maximum IgG response (antibody titer >0.200 O.D.) was achieved at day 10.0 and 62.3 in the clinical rabies and non-clinical rabies cases, respectively (p = 0.0429). No difference in IgM or IgG time to seroconversion or average maximum IgM level was observed between neck versus footpad inoculation groups. No viremia or viral shedding was detected by PCR or viral isolation during the observation period, including within the two symptomatic animals three days after disease onset. Tissue sections examined were unremarkable for inflammation or other histologic signs of rabies within the asymptomatic animal. Similarly none of the brain sections exhibited immunoreactivity for rabies virus antibody.

**Discussion:**

This study demonstrates there is no difference in time to immune response between inoculation sites and distance to the brain; however, immune response tends to be more rapid in cases of clinically apparent disease and prolonged in cases infected at sites further from the brain.

**Conclusions:**

Our findings support the hypothesis that a viremic state for rabies does not exist in the New World Monkey, *Aotus nancymaae*, and it appears that this species may be refractory to infection. The species does provide a suitable model to assess post infection immune responses. Additional studies that address the limitations of sample size, length of observation, and lack of measurable infection should be conducted.

## Background

Rabies virus, a rhabdovirus of the genus *Lyssavirus*, causes an acute fatal encephalomyelitis. All mammals are susceptible to the disease, but this may be influenced by the viral strain. Worldwide there are an estimated 24,000 to 90,000 human deaths per year, primarily in developing countries, with the largest burden occurring in Asia and Africa [1]. The primary route of transmission has long been established as contact with saliva from an infected animal, usually through a bite or scratch. The virus spreads by replicating its RNA using a reverse transcriptase and traveling primarily via the peripheral nervous system (PNS) to access the central nervous system (CNS) and numerous other organs [2]. The incubation period in humans ranges from 3–12 weeks but is thought to be as long as 7 years, depending on wound severity, wound site in relation to nerve supply and distance from the brain, and amount and strain of the virus [3]. The disease manifests as paresis and paralysis, convulsions, and ultimately death, usually due to respiratory paralysis. Wild and domestic animals can serve as reservoirs for the virus. In developing countries, dogs remain the principal reservoir [1] although contact with infected bats is becoming more common [4-9]. Six cases of human rabies spread through marmosets reared as pets occurred in Brazil from 1989–1996. [10] In the Brazilian state of Ceará, an average of 25 persons per month seek rabies post-exposure prophylaxis for marmoset and other non-human primate (NHP) bites [11,12]. During 2004–2006 the National Institute of Health in Peru reported six cases of human rabies from contact with monkeys, which are often kept as pets [13].

In this context, rearing of wild animals such as monkeys is a growing concern by the Peruvian Ministry of Health for rabies control. There is currently no established protocol or recommendation regarding pre-exposure rabies immunization of wild fauna outside of its natural habitat [14]. Appropriate rabies prophylaxis should be evaluated and considered for such animals that are often maintained outside as pets where exposure to terrestrial wildlife carnivores or bats is exceedingly possible and most likely unwitnessed [15]. Preventive therapy could potentially preclude human cases and the needless sacrifice of animals, many of which are protected by the Convention on International Trade in Endangered Species of Wild Fauna and Flora (CITES). However, an incubation period and a protective level of rabies neutralizing antibody has not been established for non-human primates, including New World species, nor has a vaccine product been licensed. Little is known about rabies virus infection in species of New World NHP, rather the majority of work in non-human primates has been conducted in Old World NHP, specifically in Rhesus monkeys. New World NHP, *Aotus nancymaae*, commonly known as owl monkeys, potentially provide a useful preclinical safety and immunogenicity model [16]. These animals have been shown to be useful as animal models for various other pathogens.

There has been controversy on whether a viremia exists with rabies infection in other animal species, but this hypothesis has yet to be tested in New World NHP. Reports of infection in other mammals seem to suggest viremia does not occur in natural infection [17,18] making the presence of a viremic state among human cases a contested issue [19]. In a study of rabies infected mice, viremia was detected in 84% of cases that developed clinical illness 11–16 days post-inoculation [20]. In addition, 52% of these clinically ill mice had detectable neutralizing antibody, partially refuting the claim that neutralizing antibody completely masks viremia [21]. Viremia has also been detected in the leucocytes of clinically ill rabbits [22].

Unlike other animal studies, the frequency of salivary gland infection in naturally-infected New World NHP is unknown. *Ante mortem* diagnosis of rabies has been reported in humans by using PCR for virus detection in but this is yet to be evaluated in monkeys [23-25]. While traditional screening tests such as rabies tissue culture inoculations tests and mouse inoculations tests gave negative results, RT-PCR was able to detect virus in saliva [24]. One study demonstrated that 5 out of 9 saliva samples from human cases were RT-PCR positive for rabies [23]. An additional study using nested RT-PCR detected virus in 9 saliva samples from 24 cases. This particular study showed that the nested RT-PCR was more sensitive than conventional RT-PCR techniques (75% sensitivity versus 37%) and is a useful *ante mortem* diagnostic tool [25].

The purpose of this study is to use *A. nancymaae* as an animal model to 1) observe the incubation period of the virus in this species, 2) determine the presence of a viremic state after infection, and 3) evaluate if PCR on saliva is an effective *ante mortem* diagnostic method. The results of these evaluations will be used in future studies to determine the effectiveness of various rabies vaccines in New World NHP and establish a protocol for these and related species. Live virus challenge for candidate vaccine testing presents advantages to simultaneously test the hypothesis that a viremic state does not exist in New World NHP and to explore the development of an effective *ante mortem* diagnostic test.

## Results

### Clinical disease and mortality

All monkeys were antibody negative for rabies virus by ELISA and without clinical rabies prior to the study challenge. Two of eight monkeys exhibited classic clinical rabies (neurologic paresis, convulsions, and wild, irrational behavior) on average 11.5 days post-inoculation. Animal 1600 developed clinical rabies 10 days post-inoculation and was euthanized on day 12. Animal 1938 developed clinical rabies 13 days post-inoculation and was euthanized on day 15. Both subjects were female and inoculated in the neck. The remaining six monkeys exhibited no signs or symptoms of clinical rabies and were euthanized at the termination of the study on day 134 post-inoculation.

### Virus detected by PCR

Results of the serum and saliva RT-PCR were negative for all monkeys before inoculation and throughout the observation period post-inoculation. Furthermore, no viral shedding was observed in either of the clinically ill monkeys during the three day observation period following onset of symptoms.

### Antibody response to rabies virus inoculation

**Figure 1:**
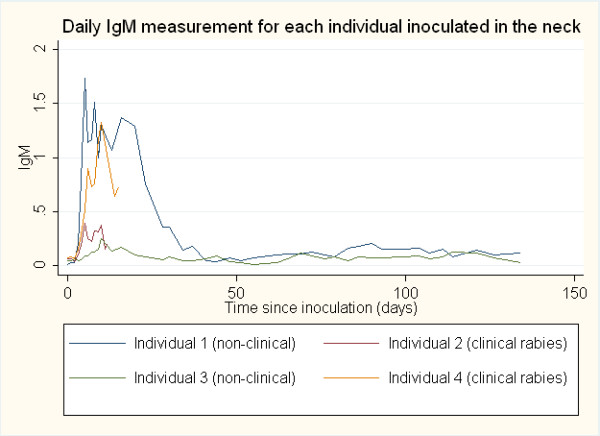
ELISA O.D. measurements for daily IgM response in individuals inoculated in the neck.

**Figure 2:**
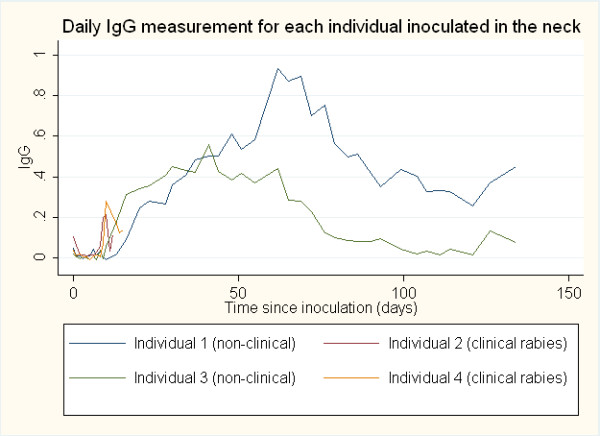
ELISA O.D. measurements for daily IgG response in individuals inoculated in the neck.

**Figure 3:**
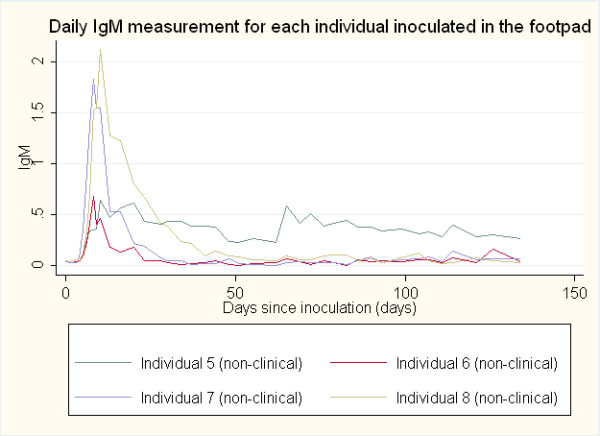
ELISA O.D. measurements for daily IgM response in individuals inoculated in the footpad.

**Figure 4:**
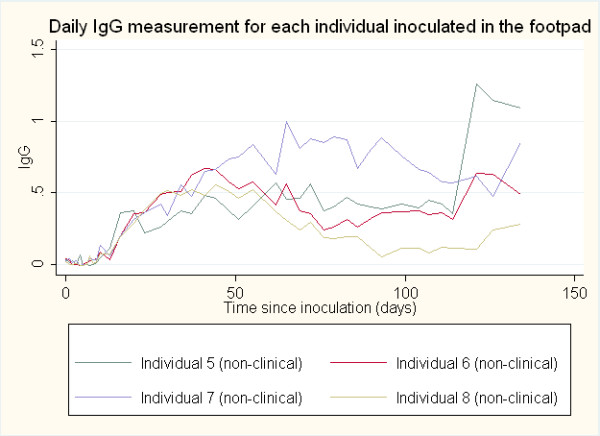
ELISA O.D. measurements for daily IgG response in individuals inoculated in the footpad.

Anti-rabies antibody responses by IgM and IgG ELISA optical density (O.D.) were compared between the clinical rabies cases and non-clinical rabies cases and between sites of inoculation (neck and footpad). The average onset time for an antibody response was determined to be the first day post-inoculation for which antibody titer (>0.200 O.D.) was detected. The daily IgM and IgG antibody responses post-inoculation in both the neck and the footpad inoculation sites are represented graphically in Figures 1, 2, 3 and 4. The day of IgM and IgG seroconversion was similar for site of inoculation (p = 0.2338 and p = 1.0000, respectively). Likewise, no variation between the peak antibody titer for IgM (p = 0.5329) and IgG (p = 0.1081) was evident between the two groups. The average onset time for an IgM response in the clinical rabies cases was 4.0 days versus 6.0 days in the non-clinical rabies cases (p = 0.1692), while the average onset time for an IgG response in each group was 10.0 and 16.7, respectively (p = 0.0614). The average maximum antibody responses occurred on day 7 in the clinical rabies cases and day 8 in the non-clinical rabies cases (p = 0.7188) for IgM and days 10.0 and 62 in the clinical rabies and non-clinical rabies cases, respectively (p = 0.0429) for IgG.

### Pathology

#### Histology

Sections available for histological analysis were brain (6/6), injection site (6/6), heart (5/6), and peripheral nerve (2/6). All sections examined were histologically unremarkable. There was neither inflammation nor Negri bodies detected in brain tissue. Likewise, there was no inflammation identified in the tissues of the injection sites or the peripheral (sciatic) nerves.

#### Immunohistochemistry

None of the brain sections exhibited immunoreactivity for rabies virus antibody. Canine cerebellum, positive for rabies on IFA, was used as a positive control. Canine sections exhibited multifocal, intracytoplasmic immunoreactivity of numerous neurons (data not shown).

## Discussion

The objectives we sought to determine in this study were the length of the incubation period, if a viremic state exists, and if PCR of saliva is an effective *ante mortem* diagnostic tool after rabies virus infection in night owl monkeys (*Aotus nancymaae*). The average incubation period of rabies was observed as 11.5 days for the two monkeys that developed clinically apparent disease. However, the study was terminated at 134 days (4.5 months) post-inoculation, at which time the remaining six study subjects did not have clinically apparent disease. Based on this information, it is possible the incubation period for infection in the *A. nancymaae* is greater than our termination point.

The presence of viral RNA in blood and saliva post-inoculation might suggest viremia and viral shedding occurs, respectively, in *A. nancymaae* and serve as an effective *ante mortem* diagnostic test; however, no viremia or viral shedding was detected in any of the study subjects. The PCR assay used in this study is highly sensitive and specific for the detection of rabies viral RNA [26]. Viral shedding in salivary sections presumably should occur irrespective of viremia, as rabies virus spread to glandular tissue is unlikely to occur via blood or epithelial cells. Previous studies have established that even with direct rabies virus inoculation, salivary gland infection is dependent on centrifugal neuronal spread and preceding CNS involvement [27]. We would not expect to measure viral shedding from salivary glands without simultaneous CNS involvement [27,28]. Our study results for the two clinical cases show that there was no detection of viral shedding pre-onset or post-onset of clinical symptoms; however, observation was only continued for three days post-onset of symptoms in the clinically ill monkeys. In contrast, viral shedding was detected in a 55-year old patient in the U.K. using RT-PCR, but this was 4 days post-onset of symptoms [24]. Also, RT-PCR successfully detected rabies virus in 5 saliva samples from 28 cases after the onset of symptoms [23]. Therefore, clinical cases may have progressed too rapidly to allow sufficient time for salivary gland infection prior to euthanasia. Other possible explanations for the lack of viral shedding include a failure of CNS involvement and centrifugal neuronal spread in non-clinical cases, inability of glandular cells to support infection, and immune response in these monkeys. Despite the development of serum neutralizing antibody, an inoculum dose-related factor may also be a possibility. Studies in foxes, dogs, and mice indicate high inoculum doses cause rapidly progressing infections leading to death prior to salivary gland infection [28], but this differs from our study as there were six cases without clinical rabies or CNS involvement.

For both the lack of viremia and viral shedding, viral neutralization, interference, and restricted infection to the site of inoculation by interferons or other non-specific immune mechanisms may have been factors [29]. Although these findings suggest there is no viremia or viral shedding of rabies virus in clinically ill *Aotus nancymae*, it is difficult to make a conclusion with such a small sample size since only two monkeys exhibited clinical symptoms. Interestingly, this study can be used to observe the time course of immunologic action between inoculation sites with different distances from the CNS. The clinically ill rabies cases were inoculated in the neck and exhibited a larger and more rapid IgG antibody response compared to the non-clinical rabies cases. Clinically apparent disease could be caused by the brain’s virus-specific T-cell response that fights against viral entry into the blood brain barrier of the monkey [30]. Since the clinically ill cases exhibited a greater response more quickly, this could simply be indicative of the virus gaining access to the CNS more rapidly. In a study challenging mice with either an avirulent or virulent strain of rabies, the antibody response was higher after seven days in the avirulent strain group, which did not develop clinical symptoms [31]. The prompt induction of neutralizing antibodies, inflammation and the inhibition of viral spread are necessary for recovery from rabies virus [32]. Once in the CNS, the virus continually replicates and leads to the onset of classical rabies symptoms [27]. Therefore, at the point of detection of high antibody titers, it would be too late to clear the virus. However, if this is the case, antibody responses in the neck inoculated monkeys that successfully overcame the rabies challenge should have also been high earlier; this suggests the influence of some other factor. The overall pattern of antibody response for the non-clinical group did not vary significantly; however, the footpad inoculated group tended to have a more robust initial IgM response and a prolonged maximum IgG response compared to the neck inoculated group. The rate of antibody response seems more important than the magnitude. A possible explanation could be that in the footpad inoculated group, the slowly developing antibody response allowed sufficient time to develop antibodies with greater avidity and counter virus in relation to its replication rate preventing it from entering the brain, essentially preventing clinical disease. This explanation is further supported by the fact that none of the brain tissue sections in the non-clinically ill cases exhibited immunoreactivity for rabies virus or histological evidence of inflammation or Negri bodies. With rabies virus infection in humans, Negri bodies are most commonly found in the hippocampus, pyramidal cells of the cerebral cortex, and Purkinje cells of the cerebellum [3]. Unfortunately, brain sections from the clinically ill rabies cases were not available for histologic and immunoreactivity analysis for comparison.

Serological testing did provide predictive value into the onset of disease. However, serological testing is of limited value because seroconversion occurs late in the course of the disease [33]. Studies in different animal models such as mice have shown that antibodies can be detected in sera [20], but this response requires the collaboration of inflammatory responses from B-cells and T-cells such as IFN-gamma [30]. Skunks experimentally-infected intramuscularly with wild-type rabies developed a detectable neutralizing antibody response 7–12 days post- inoculation but not without CNS involvement [27]. One study has shown that virus-neutralizing antibodies for rabies can be developed around 122 days after vaccination in capuchin monkeys using suckling mouse brain rabies vaccine [34]. Virus-neutralizing antibody detected in the serum can only really indicate exposure to the rabies virus; the outcome of the invasion relies on a complex interplay of viral and host factors [35]. Antibody immune response following viral inoculation relies on the replication of virus in animal tissue, in contrast to response from high levels of viral particles in commonly used inactivated vaccines [29]. However, serum neutralizing antibodies in our study were likely in response to viral antigen in the original inoculum since immunoflorescence was not detected in any pathology tissue, including the sites of infection. Extraneuronal infection in muscle is short lived, being eliminated by immune response [27,28]. This may explain the negative immunofluorescence findings at tissue inoculation sites since pathology testing was not done until 134 days post inoculation.

## Conclusion

The antibody responses of eight challenged *Aotus nancymaae*, owl monkeys, were successfully observed. Understanding the natural immune response to rabies antigen can facilitate future development of prophylactic vaccination [36]. This study suggests a viremic state does not exist with rabies infection in New World NHP; however, more studies are needed to develop *ante mortem*, practical diagnostics for rabies infection. The lack of viral shedding in experimentally-infected owl monkeys is yet to be explained and more importantly does not necessarily indicate salivary excretions are noninfectious. Owl monkeys may be able to be reproducibly infected with rabies virus but does not serve as a suitable model for infection and immunogenicity for the evaluation of candidate vaccines against rabies. It is also notable that 6 of the 8 challenged monkeys survived infection, at least up to 134 days post inoculation. One study has previously shown that other species may have evidence of rabies infection and clearance of the virus without clinical symptoms of disease [37], although this may be explained by a less virulent strain of the virus [3]. There is an inherent risk in generalizing results between different animal reservoirs as the complete picture of how rabies virus operates largely remains a mystery in many mammal species. Additional studies that address the limitations of sample size, length of observation, and lack of measurable infection should be conducted to better understand the natural history of rabies in New World NHP species.

## Methods

### *Aotus nancymaae*, New World owl monkeys

This animal study was approved by the Naval Medical Research Unit SIX (NAMRU-6) Institutional Animal Care and Use Committee and the Department of the Navy Bureau of Medicine and Surgery. Owl monkeys (*Aotus nancymaae*) were obtained from the Instituto Veterinario de Investigaciones Tropicales y de Altura, Universidad Nacional Mayor de San Marcos (San Marcos University), Peru and approved for use by the attending veterinarian. NAMRU-6 is a facility accredited by the Association for Assessment and Accreditation of Laboratory Animal Care, International; therefore all husbandry and experimental procedures were conducted in compliance with the Animal Welfare Act and The Guide for the Care and Use of Laboratory Animals [38]. All animals were singly housed in standard metal cages with nest boxes, perches, and manipulanda. A commercially formulated monkey diet (New World Primate Diet 8794 N, Harlan Teklad, Madison, WI) was provided daily and supplemented with small quantities of fresh fruit and monkey biscuits. Distilled water was provided ad libitum. Animals were provided a reverse 12:12-hour light: dark cycle that was offset from the normal day so that monkeys could be observed during their active time. The eight *A. nancymaae* monkeys randomly selected for this study were greater than 1-year age, had a weight greater than 1000 g, and showed negative tuberculosis tests within the 6 months preceding the study. Each animal was individually identified by a tattoo placed on the abdomen.

Animals were anesthetized with ketamine hydrochloride (10 mg/kg) injected intramuscularly in the caudal thigh (26 gauge, ¾-1 inch needle). This was performed for restraint during procedures such as viral inoculation, blood withdrawal, and cheek swab. Blood samples were collected from either the femoral or saphenous vein using a 26 gauge ½ inch needle and cheek swabs were collected using a sterile cotton swab rubbed against the inner surface of the cheek to obtain saliva. Animals were humanely euthanized by injection of T-61 (0.5 ml/Kg BW) IV or intracardiac using a 23 gauge, 1 inch or 21 gauge, 1 ½ inch needle after being anesthetized for restraint.

### Virus

Rabies virus used for this study was derived from rabies vaccine strain, (Pasteur)/V-13R. Viral inoculum was prepared from a cell culture lysate and clarified by centrifuge at 800 g.

### Animal challenge

A total of 8 monkeys were randomly assigned to two groups (n = 4) for inoculation intramuscularly (IM), either in the footpad or the neck, to account for differences due to wound site in relation to nerve supply and distance from the brain. Each monkey received 1.0 × 10^5^ pfu of rabies virus from 0.2-0.4 ml of viral inoculum. Group I animals were inoculated IM in the neck musculature. Group II animals were inoculated in the plantar surface of the right foot.

A daily 0.8 ml blood sample and cheek swab were collected starting 24-hours after inoculation and continued for 10 days. Baseline samples were collected at day −7 (± 3 days). After the initial 10 days post-inoculation, each blood sample and cheek swab were collected every 3 days until the onset of clinical symptoms. At the onset of symptoms, a 0.8 mL blood sample was taken and repeated daily for two days. On the third day of symptoms, a terminal 20 ml blood sample was collected and the monkey was euthanized.

To assess serologic response and viremia, blood samples were analyzed for serum neutralizing antibody titers by ELISA and for viremia by RT-PCR and viral isolation, followed by RT-PCR and sequencing. Cheek swabs were tested for rabies virus by RT-PCR and viral isolation.

### IgM and IgG ELISA

Monkey sera were analyzed for anti-Rabies IgM or IgG antibodies using an in-house ELISA. Briefly, the ELISA test uses antihuman antibody coated plates where each serum sample is added at a concentration of 1/100. These are then incubated at 37°C for 60 minutes with viral antigen. After washing, hyperimmune mouse ascitic fluid (HMAF) is added and incubated and washed again. HRP labled goat anti-mouse IgG + M (H + L) conjugate is then added to each sample, incubated and washed. Samples are read at 450 nm.

### RT-PCR

RNA viral extraction was performed from monkey sera diluted with Trizol following the manufacturer protocol. 5ul of RNA plus 1.25ul primers 1066 (10 mM) were added in a PCR tube. The solution was heated to 90°C for 2 min and cooled on ice for 5 min. 43.75 ul of RT-PCR master mix was added containing 0.25ul of primers 1066 (40 mM) and 1.25ul of primer 304 (20 mM); 5ul of 10X PCR buffer with MgCl2; 4ul of dNTPs 2.5 mM; DTT (1 M); 0.4ul of AMV reverse transcriptase (15U/ul); 0.4ul of Taq polymerase (5U/ul); and 32.2ul of Nuclease free water. The PCR conditions were: 42°C 90 min; 94°C x 3 min following of 45 cycles of 94°C x 30 sec; 37°C x 40 sec; 72°C x 90 sec; and a extension of 72°C x 10 min; kept at 4°C. 8 ul of RT- PCR were used for electrophoresis on 2% agarose gels and visualized by ethidium bromide staining.

### Viral isolation

Virus from blood and saliva samples was prepared for isolation in Vero cells. The inoculums were allowed to absorb at 37°C for 60 min in a 5% CO2 humidified incubator and kept in maintenance media (2% fetal bovine serum - FBS) in a plate or flask. Cells were incubated at 37°C in 5% CO2 for 10 days. Cells were observed daily for cytopathic effects (CPE). After 10 days, an immunofluorescence assay (IFA) for FITC Anti-rabies Monoclonal Globulin was conducted.

### Pathology

#### Histology

Routine histological analysis was conducted on tissue sections of whole brain, injection site, heart, and peripheral (sciatic) nerve. Three sections of brain from each non-human primate were prepared and examined, which included the hippocampus, pyramidal cells of the cerebral cortex, and Purkinje cells of the cerebellum. Brain tissue was taken at 25 mm and 5 mm caudal to and 10 mm cranial to the anterior commissure. Tissues were routinely processed, sectioned at 6 μm and stained with H&E.

#### Immunohistochemistry

Immunohistochemical detection of rabies virus (glycoprotein of rabies virus) was performed on sections of formalin fixed, paraffin embedded blocks of non-human primate cerebrum. Slides were deparaffinized using xylene followed by graded baths of ethanol. A DAKO Autostainer Plus Universal Staining System (DAKO, Carpenteria, CA) was used for the automated immunohistochemical staining. Antigen retrieval was performed using Citra (BioGenex, San Ramon, CA) for 30 minutes. Mouse monoclonal antibody to rabies virus (Abcam, Cambridge, MA) was used at a dilution of 1:500 and incubated overnight at 4 C. LSAB2-HRP dual anti-rabbit/anti-mouse (DAKO, Carpenteria, CA) was applied ready to use as a secondary antibody for 30 minutes at room temperature. The chromogen applied was 3, 3’ Diaminobenzidine (DAKO, Carpenteria, CA). The sections were counterstained with Hematoxylin (DAKO, Carpenteria, CA) and then coverslipped.

### Data analysis

All data analysis was performed using Stata/SE 11.1 for Windows (StataCorp LP, College Station, TX). Life tables for survival analysis were used for evaluation of seroconversion levels of antibodies. Wilcoxon rank-sum non-parametric test was used to assess average time to seroconversion and maximum antibody levels between clinically ill and non-clinically ill cases as well as to test differences in the site of inoculation. Significance levels were considered as the standard <0.05.

## Abbreviations

PCR: Polymerase Chain Reaction; ELISA: Enzyme Linked Immosorbent Assay; OD: Optical Density; PFU: Plaque Forming Units; CPE: Cytopathic Effects; IFA: Immunofluorescence Assay; IgM: Immune globulin M; IgG: Immune globulin G; CNS: Central Nervous System; PNS: Peripheral Nervous System; IM: Intramuscular.

## Competing interests

We are uniformed service members or an employee of the U.S. Government and this work was done as part of our official duties. Title 17 U.S.C. §105 provides that ‘Copyright protection under this title is not available for any work of the United States Government.’ Title 17 U.S.C. §101 defines a U.S. Government work as a work prepared by a military service member or employee of the U.S. Government as part of that person’s official duties. The views expressed in this article are those of the authors and do not necessarily reflect the official policy or position of the Department of the Navy, Department of Defense, nor the U.S. Government. This work wasfunded by the Department of Defense Global Emerging Infectious Surveillance and Response Program, grant 847705 82000 25 GB B0016.

## Author contributions

JMM and DEB devised and designed the study. DEB supervised all animal experiments. CG and TJK carried out immunoassays and molecular testing. TJS performed all pathology exams. EJR and GSM analyzed the data and drafted the manuscript. All authors read, edited and approved the final manuscript.

## References

[B1] WHO, W Expert Consultation on Rabies 2005 WHO Technical Report Series: first report 931 16485446

[B2] Duenas A Isolation of rabies virus outside the human central nervous system J Infect Dis 1973 127 6 702 704 4707313 10.1093/infdis/127.6.702

[B3] Jackson AC Rabies virus infection: an update J Neurovirol 2003 9 2 253 258 12707856 10.1080/13550280390193975

[B4] Belotto A Overview of rabies in the Americas Virus Res 2005 111 1 5 12 15896398 10.1016/j.virusres.2005.03.006

[B5] Favi M First case of human rabies in chile caused by an insectivorous bat virus variant Emerg Infect Dis 2002 8 1 79 81 11749754 10.3201/eid0801.010108 PMC2730271

[B6] Gomez-Benavides J Outbreak of human rabies in Madre de Dios and Puno, Peru due to contact with the common vampire bat, Desmodus rotundus AmJTrop Med Hyg 2007 77 5_Suppl 150 199

[B7] Salmón-Mulanovich G Human rabies and rabies in vampire and nonvampire, southeastern Peru, 2007 Emerg Infect Dis 2009 15 8 3 10.3201/eid1508.081522 PMC2815962 19751600

[B8] Schneider MC Rabies transmitted by vampire bats to humans: an emerging zoonotic disease in Latin America? Rev Panam Salud Publica 2009 25 3 260 269 19454154 10.1590/s1020-49892009000300010

[B9] Suzan G Modeling hantavirus reservoir species dominance in high seroprevalence areas on the azuero peninsula of panama AmJTrop Med Hyg 2006 74 6 1103 1110 16760528

[B10] Andrade MC Immune response produced by anti-rabies vaccines in marmosets(Callithrix sp) Rev Soc Bras Med Trop 1999 32 5 533 540 10881088 10.1590/s0037-86821999000500011

[B11] Favoretto SR Rabies virus maintained by dogs in humans and terrestrial wildlife, Ceara State, Brazil Emerg Infect Dis 2006 12 12 1978 1981 17326958 10.3201/eid1212.060429 PMC3291349

[B12] Favoretto SR Rabies in marmosets (Callithrix jacchus), Ceara, Brazil Emerg Infect Dis 2001 7 6 1062 1065 11747745 10.3201/eid0706.010630 PMC2631923

[B13] INS Vargas Herrera J Informe Especial La Rabia en el Perú 2004–2006 2006 Lima: Instituto Nacional de Salud 33

[B14] NASPHV Compendium of Animal Rabies Prevention and Control, 2011 2011 Atlanta: Morbidity and Mortality Weekly Report (MMWR) 1 14 60 22052042

[B15] Kenny DE Exposure of hooded capuchin monkeys (Cebus apella cay) to a rabid bat at a zoological park J Zoo Wildl Med 2001 32 1 123 126 12790408 10.1638/1042-7260(2001)032[0123:EOHCMC]2.0.CO;2

[B16] Jones FR The New World primate, Aotus nancymae, as a model for examining the immunogenicity of a prototype enterotoxigenic Escherichia coli subunit vaccine Vaccine 2006 24 18 3786 3792 16343702 10.1016/j.vaccine.2005.07.029

[B17] Constantine DG Absence of prenatal infection of bats with rabies virus J Wildl Dis 1986 22 2 249 250 3520031 10.7589/0090-3558-22.2.249

[B18] Sitprija V Does contact with urine and blood from a rabid dog represent a rabies risk? Clin Infect Dis 2003 37 10 1399 1400 14583881 10.1086/379130

[B19] Burne JC Viraemia in rabies Lancet 1970 1 7639 195 196 4189278 10.1016/s0140-6736(70)90444-7

[B20] Lodmell DL Dimcheff DE Ewalt LC Viral RNA in the bloodstream suggests viremia occurs in clinically ill rabies-infected mice Virus Res 2006 116 1–2 114 118 16242805 10.1016/j.virusres.2005.09.004

[B21] Sims RA Allen R Sulkin SE Studies on the pathogenesis of rabies in insectivorous bats. III. Influence of the gravid state J Infect Dis 1963 112 17 27 13977575 10.1093/infdis/112.1.17

[B22] Baratawidjaja RK Morrissey LP Labzoffsky NA Demonstration of vaccinia, lymphocytic choriomeningitis and rabies viruses in the leucocytes of experimentally infected animals Arch Gesamte Virusforsch 1965 17 2 273 279 4956729 10.1007/BF01267911

[B23] Crepin P Intravitam diagnosis of human rabies by PCR using saliva and cerebrospinal fluid J Clin Microbiol 1998 36 4 1117 1121 9542950 10.1128/jcm.36.4.1117-1121.1998 PMC104702

[B24] Smith J Case report: rapid ante-mortem diagnosis of a human case of rabies imported into the UK from the Philippines J Med Virol 2003 69 1 150 155 12436491 10.1002/jmv.10253

[B25] Nagaraj T Ante mortem diagnosis of human rabies using saliva samples: comparison of real time and conventional RT-PCR techniques J Clin Virol 2006 36 1 17 23 16504574 10.1016/j.jcv.2006.01.009

[B26] Hughes GJ Evaluation of a TaqMan PCR assay to detect rabies virus RNA: influence of sequence variation and application to quantification of viral loads J Clin Microbiol 2004 42 1 299 306 14715769 10.1128/JCM.42.1.299-306.2004 PMC321704

[B27] Charlton KM Casey GA Campbell JB Experimental rabies in skunks: immune response and salivary gland infection Comp Immunol Microbiol Infect Dis 1987 10 3–4 227 235 3427891 10.1016/0147-9571(87)90033-6

[B28] Charlton KM Casey GA Campbell JB Experimental rabies in skunks: mechanisms of infection of the salivary glands Can J Comp Med 1983 47 3 363 369 6357414 PMC1235954

[B29] Oliveira AN Immune response in cattle vaccinated against rabies Mem Inst Oswaldo Cruz 2000 95 1 83 88 10656710 10.1590/s0074-02762000000100013

[B30] Hooper DC Collaboration of antibody and inflammation in clearance of rabies virus from the central nervous system J Virol 1998 72 5 3711 3719 9557653 10.1128/jvi.72.5.3711-3719.1998 PMC109593

[B31] Irwin DJ Basis of rabies virus neurovirulence in mice: expression of major histocompatibility complex class I and class II mRNAs J Neurovirol 1999 5 5 485 494 10568885 10.3109/13550289909045377

[B32] Warrell MJ Warrell DA Rabies and other lyssavirus diseases Lancet 2004 363 9413 959 969 15043965 10.1016/S0140-6736(04)15792-9

[B33] Schuller E IgM and IgG antibody responses in rabies encephalitis Ann Microbiol (Paris) 1979 130A 3 365 372 484994

[B34] Passos EC Inactivated suckling mouse brain rabies vaccine provides short-term immunity in capuchin monkeys (Cebus apella) J Zoo Wildl Med 2001 32 1 55 57 12790394 10.1638/1042-7260(2001)032[0055:ISMBRV]2.0.CO;2

[B35] Baer GM The natural history of rabies1991 1991 Boston: CRC

[B36] Johnson N Cunningham AF Fooks AR The immune response to rabies virus infection and vaccination Vaccine 2010 28 23 3896 3901 20368119 10.1016/j.vaccine.2010.03.039

[B37] East ML Regular exposure to rabies virus and lack of symptomatic disease in Serengeti spotted hyenas Proc Natl Acad Sci U S A 2001 98 26 15026 15031 11742089 10.1073/pnas.261411898 PMC64977

[B38] Council NR Guide for the Care and Use of Laboratory Animals1996 1996 Washington, D.C: National Academy Press 125

